# The Texas State Lunatic Asylum

**Published:** 1889-12

**Authors:** 


### THE TEXAS STATE LUNATIC ASYLUM.

We are in receipt of the Report of the Superintendent of the State Lunatic Asylum for 1889. It is a very interesting document and shows a satisfactory status under the present administration. On 31st of October, 1888, there were in the asylum 585 inmates; admitted during the year, 160; discharged during the year, 89, of whom 45 were reported cured, and 36 much improved; 4 taken home by friends, incurable. On hand October 31, 1889, 621—the largest number of patients present at any one time since the founding of the institution. The per capita cost of maintaining them was $170.30 a year, or $3.30 a week.

During the year 745 patients were treated, with 24 deaths; a ratio of 3.22. It is a remarkable and very creditable fact that this is the lowest average mortality ever recorded in the history of the asylum,—the highest being in 1881,—11.59. During the three years of Dr. Dorset's administration the yearly average mortality has steadily decreased, it having been, in 1887, 5.61; in 1888, 4.95, and in 1889, 3.22; the number of patients treated being respectively, 730, 707, 745, and the corresponding deaths being 41, 35, 23.

The institution is largely self-supporting; i. e., there were produced on the farm, 5000 bushels of corn, 10,215 pounds of pork, besides vegetables and other produce, while the work done in the broom factory and mattress shop is very creditable. Heretofore, the superintendent says, all the brooms and mattresses were purchased. The superintendent of this department, Mr. Tom Anderson, is blind, and learned his trade in the blind institute here in Austin, and his only assistant is a patient of the asylum.

The superintendent bears testimony to the ability, efficiency and zeal of his staff of assistants, and says, they have with ceaseless care performed their several duties as faithful servants of the State.

